# Dynamic strain evolution in an optically excited Pt thin film

**DOI:** 10.1063/5.0067770

**Published:** 2021-11-08

**Authors:** M. F. DeCamp, A. D. DiChiara, K. M. Unruh

**Affiliations:** 1 Department of Physics and Astronomy, University of Delaware, Newark, Delaware 19716, USA; 2 Advanced Photon Source, Argonne National Laboratory, 9700 S. Cass Ave., Argonne, Illinois 60439, USA

## Abstract

The structural evolution of a Pt thin film following photo-thermal excitation by 1 ps optical laser pulses was studied with a time resolution of 100 ps over a total time period of 1 ms. Laser pulse fluences below 50 mJ/cm^2^ were insufficient to relax the residual stress state of the as-prepared film even after 10 000 pulses. In this fluence regime, a rapid initial lattice expansion and a decrease in the lattice coherence length due to ultrafast photo-thermal heating were observed. The lattice expansion reached a maximum, and the coherence length reached a minimum, 100–200 ps after excitation before monotonically decaying back to their initial values in about 1 *µ*s. Laser pulse fluences greater than 50 mJ/cm^2^ produced irreversible stress relaxation within the first 10 optical pulses. In this regime, the lattice expansion was qualitatively similar to that in the low fluence regime, except that the initial structural state was not recovered. The evolution in the coherence length, however, was more complex. Following an initial decrease similar to that observed at low fluence, the coherence length then increased to a broad maximum greater than the initial value, before recovery.

Vapor deposited thin films often exhibit residual stresses that arise from the presence of a free surface, film–substrate interactions, and non-equilibrium preparation conditions.^1^ These stresses play an important role in the formation and stabilization of a variety of thin film structures, many of which exhibit useful technological properties.^2^ As a result, the ability to control and manipulate the stress in thin films during preparation, processing, device packaging, and subsequent use is of great practical importance.

X-ray diffraction (XRD) is perhaps the most important experimental technique available for studying the structure of condensed matter. Because of the limited time resolution provided by traditional x-ray sources and detectors, however, XRD measurements have historically been restricted to the study of static or quasi-static structures. This situation has dramatically changed over the last few decades as new x-ray sources, detectors, and experimental techniques have been developed and become widely available. These advances now allow high-resolution XRD patterns to be acquired on timescales ranging from tens of femtoseconds (using x-ray plasma sources and free electron lasers) to tens of picoseconds (at third generation synchrotrons).^3^

Optical pump/x-ray probe experiments, in which a transient photo-thermal excitation is followed by a time-resolved x-ray diffraction (TRXRD) measurement, have been used to study many fundamental physical processes such as electron–phonon interactions,^4–6^ coherent acoustic oscillations,^5,7–9^ and phase transitions.^10–12^ If the excitation energy is not too large, these processes are reversible and an unlimited number of pump/probe data acquisitions can be acquired and averaged at the same sample location. If excitation leads to irreversible structural changes, either the intensity of the x-ray beam must be large enough to allow “single shot” data acquisitions or multiple diffraction patterns must be acquired and averaged at different sample locations. The high intensity x-ray pulses needed for single shot experiments are typically polychromatic and provide less angular resolution. High angular resolution requires lower intensity monochromatic x-ray pulses and averaging multiple pump/probe data acquisitions. Because of this trade-off, TRXRD studies of irreversible processes such as those associated with stress relaxation are less common than the study of reversible processes. Our interest in TRXRD studies of irreversible processes, in general,^13^ and the practical importance of gaining a better understanding of irreversible thin film stress relaxation, in particular, provided the primary motivation for the work described in this article.

The Pt thin film used in this work was prepared by a standard magnetron sputter deposition method. A single crystal Si wafer with a 1 *µ*m thick amorphous silicon oxide surface layer was used as the substrate. The measured film thickness was 37 nm based on a fit to the oscillations in the low angle x-ray reflectivity spectra.

Except for the initial film characterization measurements, all of the diffraction experiments were performed at sector 14-ID-B (BioCARS) at Argonne National Laboratory’s Advanced Photon Source (APS).^14^ The beamline and downstream mechanical and optical elements were configured to produce a “pink” beam of isolated 100 ps duration x-ray pulses with a nominal peak energy of 12 keV and a bandwidth of about 600 eV. After horizontal and vertical focusing by a Kirkpatrick–Baez mirror system, the beam was reflected from a specially designed pair of multilayer x-ray mirrors and the third order scattered beam (chosen for its optimized combination of flux and bandwidth) isolated.^15^ This beam contained about 1.5 × 10^8^ photons/100 ps pulse, a measured bandwidth of ∼70 eV, and a peak energy of 11.950 keV. Compared to the pink beam, the order of magnitude improvement in the bandwidth came at the cost of a reduction in the beam flux by a factor of 200. On the other hand, while the bandwidth was 45 times greater than that of a monochromatic beam, the x-ray flux was more than a factor of 10 greater. It is worth noting that the intrinsic line broadening due to finite size effects in nanostructured materials often prohibits taking full advantage of the very small bandwidth of monochromatic x rays. In these cases, the trade-off of a greater flux at the expense of a somewhat broader energy distribution can be particularly advantageous.

A Spectra Physics Spitfire optical laser system was used to produce 790 nm, 1 ps duration optical pump pulses. The time delay between the excitation and probe pulses was electronically controlled to within 10 ps (independent of the actual delay). A variable neutral density filter was used to vary the optical fluence on the sample from 12 to 95 mJ/cm^2^. Larger fluences produced visible sample damage. Each laser pulse was defocused to a size of 550(H) × 500(V) *µ*m^2^ at the sample, several times larger than the x-ray spot size, to ensure a relatively uniform energy deposition over the entire region probed by the x-ray pulse.

A Rayonix MX340-HS camera with an active area of 34 × 34 cm^2^ was used to collect the XRD patterns. The contents of four individual pixels in a 2 × 2 array were combined to give an effective pixel size of 88 × 88 *µ*m^2^. The detector was located 200 mm from the sample. At this distance, the camera could record scattered wavevectors up to Q = 4π sinθ/λ = 42.5 nm^−1^ over an angle of about ψ = ±75° with respect to the sample plane normal. In this geometry, the (111)/$\left(\bar{1}11\right)$/$\left(11\bar{1}\right)$ members of the {111} family and the (200)/(002) members of the {200} family of Pt diffraction peaks could be observed. The $\left(\bar{1}11\right)$/$\left(11\bar{1}\right)$ and (200)/(002) peaks were centered at about ψ = ±70° and ψ = ±55°, respectively, consistent with a near cubic lattice structure.

Previously acquired “dark” background images were numerically subtracted from each x-ray exposure and transient “hot” pixels manually removed. Radial integrations of the corrected images were then carried out at a given Q as a function of ψ in angular wedges of various sizes. The resulting 1D diffraction peaks were individually fit to a split pseudo-Voigt line shape [symmetrically positioned $\left(\bar{1}11\right)$/$\left(11\bar{1}\right)$ and (200)/(002) diffraction peaks were averaged prior to fitting in order to improve the signal-to-noise ratio]. Finally, the fitted peak positions were corrected based on the measured peak positions of a CeO_2_ powder standard. The relative angular widths of the CeO_2_ diffraction peaks were ΔQ/Q ≈ 0.006, indicating that the angular resolution of the experiment was bandwidth limited.

Prior to the TRXRD measurements, a series of static XRD measurements were carried out on the as-prepared samples and samples that had been annealed by exposing them to as many as 10 000 laser pulses. Laser annealing only produced structural changes due to stress relaxation above a pulse fluence threshold of about 50 mJ/cm^2^. Typical results for pulse fluences below and above the 50 mJ/cm^2^ threshold following 1200 laser pulses are shown in Fig. 1. The presence of an intense (111) peak and the absence of a (200) peak at ψ = 0° in the 2D diffraction image indicate a strong degree of (111) texture in both the as-prepared and laser irradiated samples [Figs. 1(a)–1(d)]. Moreover, the presence of (200) and (002) peaks at about ψ = ±55° indicates that the texture is of a fiber-type as often observed in sputter deposited FCC metal films.^16^

**FIG. 1.**
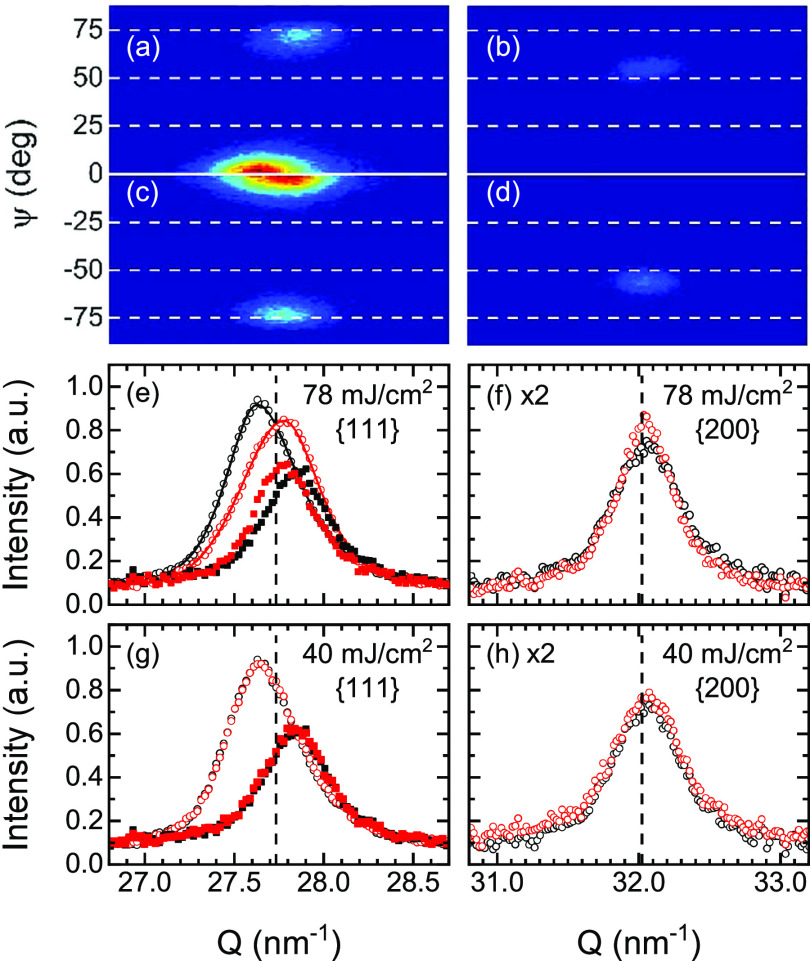
2D diffraction images as a function of Q and ψ before (a) and (b) and after (c) and (d) 1200 laser pulses at a fluence of 78 mJ/cm^2^. Radially integrated intensities before (black symbols) and after (red symbols) laser irradiation (e)–(h) at the indicated laser fluence. Open symbols correspond to the (111) or (200)/(002) peaks. Closed symbols correspond to the $\left(\bar{1}11\right)/\left(11\bar{1}\right)$ peaks. Only the fits of the (111) peaks at 78 mJ/cm^2^ are shown for clarity. The vertical dashed lines indicate the position of the bulk Pt diffraction peaks.

The d-spacings calculated from the as-prepared (111), $\left(\bar{1}11\right)$/$\left(11\bar{1}\right)$, and (200)/(002) diffraction peaks were d_111_ = 0.227 42(2) nm, $d_{\bar{1}11/11\bar{1}}=0.225\;62\left(3\right)$ nm, and d_200/002_ = 0.196 09(2) nm. In comparison to the bulk Pt lattice parameter of a_0_ = 0.392 42 nm,^17^ these values represent relative strains ɛ_hkl_ = (d_hkl_ − d_0_)/d_0_ of ɛ_111_ = +0.38% (expansion), $\varepsilon_{\bar{1}11/11\bar{1}}=-0.42\%$ (compression), and ɛ_200/002_ = +0.06% (expansion). The interpretation of these strain values is discussed below. The linewidths of the (111) “on-axis” peak at ψ = 0° and the $\left(\bar{1}11\right)$/$\left(11\bar{1}\right)$ “off-axis” peaks at about ψ = ±70° yielded lattice coherence lengths of 12.7 and 14.5 nm, respectively, indicating that the fiber axis did not extend over the entire film thickness.

As previously noted, no stress relaxation was observed until the optical fluence exceeded 50 mJ/cm^2^ (Fig. 1). Above 50 mJ/cm^2^, the (111) d-spacing irreversibly decreased and the $\left(\bar{1}11\right)$/$\left(11\bar{1}\right)$ d-spacings increased with increasing pulse fluence. For example, after 1200 laser pulses at a fluence of 78 mJ/cm^2^, the measured values of d_111_ = 0.226 19(2) nm and $d_{\bar{1}11/11\bar{1}}=0.226\;39\left(5\right)$ nm, corresponding to lattice strains of −0.16% and −0.08%, respectively, revealed a significant stress reduction with respect to the bulk [Fig. 1(e)]. The (200)/(002) d-spacing remained unchanged in both the as-prepared and laser annealed samples. Neither the film texture nor the observed crystallite size changed appreciably due to laser annealing.

In addition to the static XRD measurements described in the previous paragraphs, two sets of TRXRD experiments were also carried out. In the first instance, the previously annealed samples were studied as a function of the laser pulse energy. In the low fluence regime, the results of these measurements were identical to those on the as-prepared samples. In this case, the TRXRD patterns reveal the effects of reversible transient thermal heating in the absence of irreversible structural changes due to stress relaxation. The second set of experiments was carried out starting with an as-prepared sample. Under these conditions, the TRXRD patterns exhibited the superimposed effects of both reversible thermal heating and irreversible stress relaxation. For both sets of experiments, multiple TRXRD patterns at each time delay and sample location were averaged to obtain a usable diffraction pattern.

The results of a typical TRXRD experiment in the low and high laser pulse energy regimes are shown in Fig. 2 for the pre-annealed samples. The measured strains and linewidths begin to rise prior to Δt = 0 because the time delay was determined based on the peak positions of the x-ray and optical pulses. As a result, the leading edge of the 100 ps duration x-ray pulse starts to sample the effects of laser excitation at time delays before Δt = 0.

**FIG. 2.**
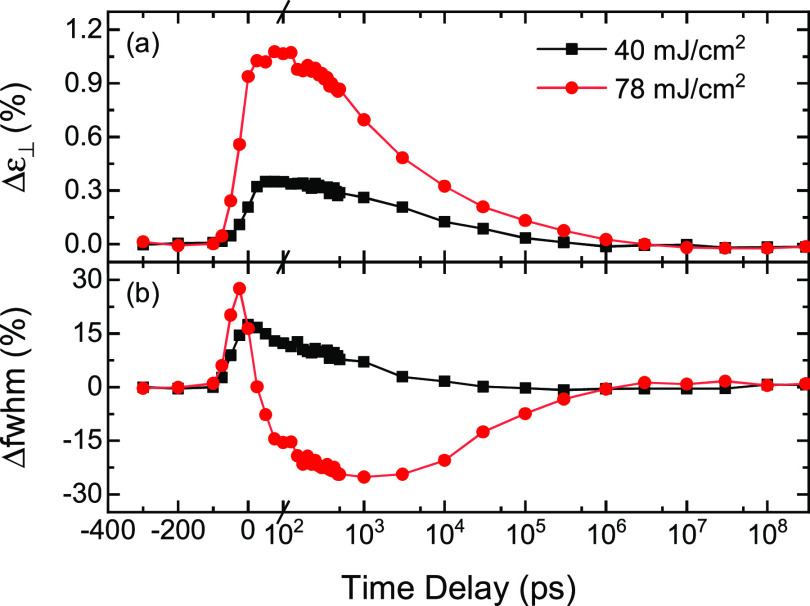
Time-resolved perpendicular strain (a) (Δɛ_⊥_) and linewidth (b) (Δfwhm) obtained from the (111) diffraction peak after 1200 laser pulses at a fluence of 40 (black squares) and 78 (red circles) mJ/cm^2^. The strains and linewidths have been individually normalized to their initial laser annealed values.

The time evolution of the lattice strain determined from the relative shift in the angular positions of the (111), $\left(\bar{1}11\right)$/$\left(11\bar{1}\right)$, and (200)/(002) diffraction peaks was qualitatively similar to that shown in Fig. 2(a) at all pulse energies, first increasing to a broad maximum between 100 and 200 ps, before monotonically decreasing back to the initial value in about 1 *µ*s. For the 78 mJ/cm^2^ pulses, the maximum measured strain was 1.1%. Assuming a bulk thermal expansion coefficient of 10^−5^ K^−1^,^18^ this strain would correspond to a lattice temperature of about 1400 K, well below the bulk melting point of 2040 K. Consistent with this temperature estimate, no evidence for melting was observed.

A number of TRXRD studies on thin metallic and insulating films have observed coherent acoustic oscillations when the duration of the laser excitation pulse is less than a few picoseconds.^5,7–9^ The period of these oscillations is material and film thickness dependent but is typically tens of picoseconds for a 50 nm thick metallic film, considerably less than the temporal resolution of our TRXRD measurement. As a result, it is not surprising that we do not observe these oscillations. We therefore attribute the initial rapid increase in the film strain shown in Fig. 2 to simple thermal expansion due to rapid heating of the film lattice as the laser energy transferred to the conduction electrons of the film decays to the lattice.

Unlike the lattice strain, the time evolution of the lattice coherence length was significantly different in the low and high intensity regimes [Fig. 2(b)]. In both cases, the linewidth reached its maximum in less than 100 ps after photo-excitation. In the low energy regime, the linewidth then monotonically decreased (and the coherence length increased) to its initial equilibrium value. Similar behavior was observed for all the diffraction peaks in the {111} and {200} families. In the high fluence regime, however, the (111) linewidth first increased and then decreased to a broad minimum below the initial value before recovering again. At a pulse fluence of 78 mJ/cm^2^, the largest measured linewidth was about 30% larger and the minimum linewidth was about 25% smaller than the initial value (corresponding to a decrease and an increase in the coherence length by a factor of about 2). A similar evolution in the linewidth was not observed for the $\left(\bar{1}11\right)$/$\left(11\bar{1}\right)$ and (200)/(002) diffraction peaks, indicating that there is a strong directional asymmetry in the dynamical response of the lattice coherence to laser excitation.

The free surface in a thin film sample requires a vanishing sample stress perpendicular to the surface. If, in addition, the averaged macroscopic in-plane stress is assumed to be isotropic, it follows that $\left\langle \sigma_{11}^{S}\right\rangle =\left\langle \sigma_{22}^{S}\right\rangle \equiv \sigma_{∥}$, $\left\langle \sigma_{12}^{S}\right\rangle =\left\langle \sigma_{21}^{S}\right\rangle =0$, and $\left\langle \sigma_{3i}^{S}\right\rangle =\left\langle \sigma_{i3}^{S}\right\rangle =0$, where i = 1, 2, or 3, the subscripts 1 and 2 refer to the in-plane axes, and the subscript 3 refers to the perpendicular axis of a coordinate system fixed to the sample (S). The resulting strains are $\left\langle \varepsilon_{11}^{S}\right\rangle =\left\langle \varepsilon_{22}^{S}\right\rangle \equiv \varepsilon_{\|}$, $\left\langle \varepsilon_{12}^{S}\right\rangle =\left\langle \varepsilon_{21}^{S}\right\rangle =0$, and $\left\langle \varepsilon_{33}^{S}\right\rangle \equiv \varepsilon_{⊥}$. For a textured thin film sample, a measurement of the diffraction strain $\varepsilon_{\psi }^{hkl}$ of an (hkl) diffraction peak as a function of ψ is given by^19–21^

$$\varepsilon_{\psi }^{hkl}=\varepsilon_{\|}\sin^{2}\psi +\varepsilon_{⊥}\cos^{2}\psi +\left\langle \varepsilon_{13}^{S}\right\rangle \sin2\psi .$$ (1)

Equation (1) allows ɛ_‖_, ɛ_⊥_, and $\left\langle \varepsilon_{13}^{S}\right\rangle$ to be determined based on at least three different measured values of $\varepsilon_{\psi }^{hkl}$. If desired, these measured strains could also be related to the elastic constants of the crystallites in the film given a grain interaction model.^20,21^ Such an analysis is beyond the scope of this paper. The maximum relative strains $\Delta \varepsilon_{hkl}^{\max}$ for the (111), $\left(\bar{1}11\right)$/$\left(11\bar{1}\right)$, and (200)/(002) diffraction peaks from the TRXRD measurements, as well as the corresponding values of ɛ_‖_, are shown in Fig. 3 as a function of the incident laser fluence. The maximum strain in both the low and high intensity regimes is approximately linear in the optical fluence with a greater slope in the high intensity regime compared to the low intensity regime. On the other hand, the calculated value of ɛ_∥_ is relatively constant in the low and high intensity regimes with different values in each. This result emphasizes the fact that the dynamical response of the film is more complex than the simple elastic behavior observed in bulk materials where one would expect the ratio of the perpendicular to the parallel stain to be constant. The correlation between the onset of irreversible stress relaxation and the change in the slope of the peak strain suggests that above a threshold fluence of about 50 mJ/cm^2^, a new region of the sample’s energy landscape becomes accessible.

**FIG. 3.**
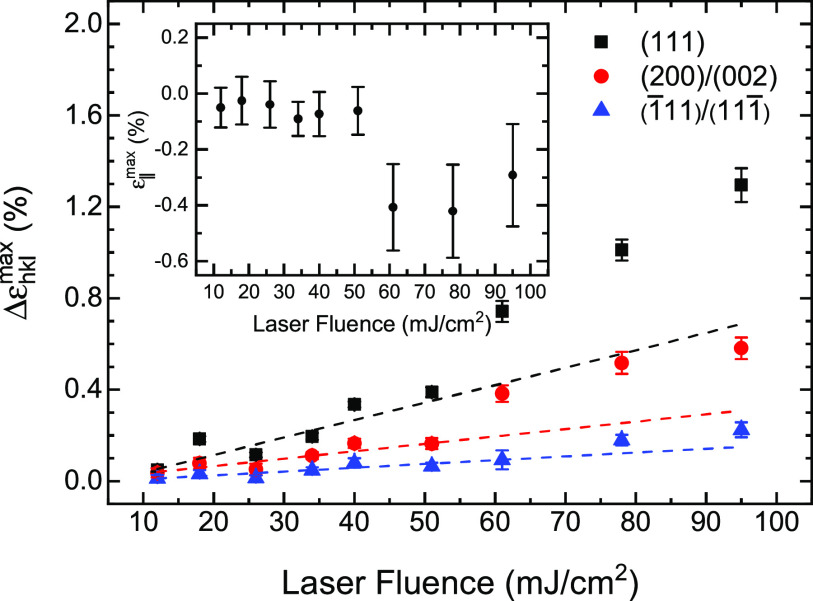
Maximum strain $\left(\Delta \varepsilon_{hkl}^{\max}\right)$ obtained from the (111), $\left(\bar{1}11\right)/\left(11\bar{1}\right)$, and (200)/(002) diffraction peaks as a function of the integrated laser fluence. The strains have been individually normalized to the initial laser annealed values for each diffraction peak. The calculated in-plane strain (ɛ_∥_) obtained from Eq. (1) is shown in the inset.

The results of a series of TRXRD experiments starting from an as-prepared sample are shown in Fig. 4. The solid red circles in Fig. 4(a) show the strain, normalized to the as-prepared/zero laser pulse state, at the indicated time delay and integrated number of 78 mJ/cm^2^ laser pulses. Only those time delays from −100 to +475 ps are shown in order to maintain a linear relationship between the time delay and the number of laser pulses (the data points at 10 and 20 laser pulses correspond to time delays of −300 and −200 ps, respectively). Each measurement was carried out at the same sample location by averaging 10 pump/probe cycles at the given time delay. The dashed line is the average of five subsequent measurements and reflects the purely reversible response of the sample after laser annealing. The behavior shown is typical of the high fluence regime. These measurements show that less than 10 laser pulses are required to produce irreversible stress relaxation and that the final relaxed state is obtained within about 50 laser pulses. The data shown in Fig. 4(a), and similar data at several different fluences (not shown), are replotted in Fig. 4(b) to emphasize the effects of laser fluence and total deposited laser energy on the film structure. In particular, the quantity $\Delta \varepsilon_{⊥}^{di\mathrm{ff}}$ represents the difference between the measured strain for an as-prepared sample at a given time delay and total number of laser pulses [e.g., the solid red circles in Fig. 4(a)] and the average strain of an annealed sample at the same time delay [dashed red line in Fig. 4(a)] after shifting both to a common origin. These results clearly show that laser fluences less than 50 mJ/cm^2^ do not produce stress relaxation, while larger laser fluences produce increasing stress relaxation.

**FIG. 4.**
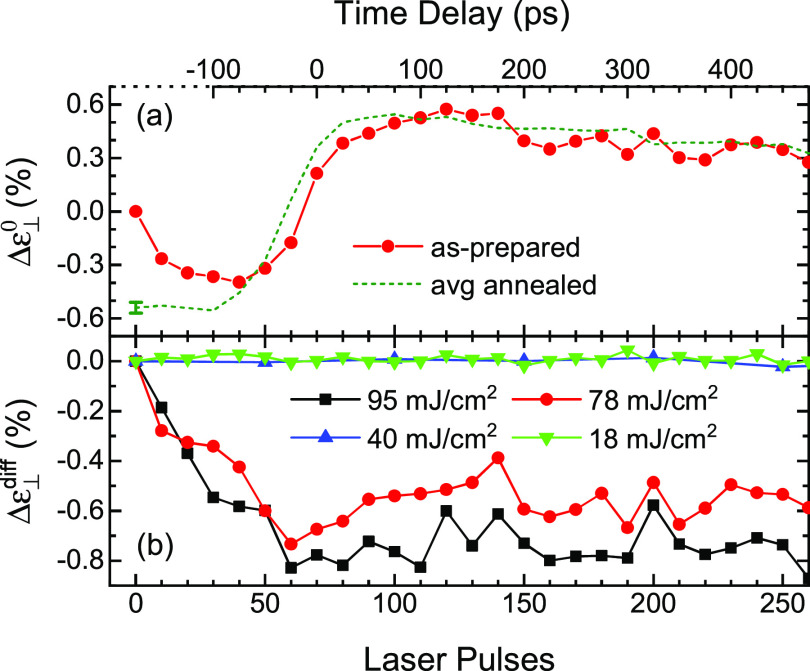
(a) Relative strain $\left(\Delta \varepsilon_{⊥}^{rel}\right)$ at each indicated time delay (note that the data points at 10 and 20 laser pulses correspond to time delays of −300 and −200 ps, respectively) and corresponding total number of 78 mJ/cm^2^ laser pulses. The solid red circles are the results starting from an initially as-prepared sample and the dark green dashed line is the average of five subsequent sets of measurements at the same sample location after laser annealing. (b) The difference between the initial as-prepared strain and the average annealed strain.

In summary, the static and dynamic evolution of a Pt thin film following photo-thermal excitation by 1 ps optical laser pulses was studied as a function of the pulse fluence and total energy deposition. These measurements revealed two distinct response regimes. For laser fluences less than 50 mJ/cm^2^, the sample response to photo-excitation was a fluence dependent reversible lattice expansion coupled with a decrease in the lattice coherence, which decayed to the initial structure after about 1 *µ*s. The maximum lattice expansion and minimum coherence length were reached 100–200 ps after the arrival of the laser pulse. Laser fluences greater than 50 mJ/cm^2^ produced irreversible structural relaxation in less than 10 laser pulses. In this regime, transient thermal heating produced a lattice expansion and decay similar to that observed in the low fluence regime, except that the initial structural state was not recovered. Unlike the low fluence regime, however, the lattice coherence length first exhibited an initial decrease (similar to that observed in the low fluence regime) followed by a subsequent increase to a value greater than the initial value before a subsequent recovery.

## Data Availability

The data that support the findings of this study are available from the corresponding author upon reasonable request.
